# Cross-sectional study of the prehospital management of adult patients with a suspected seizure (EPIC1)

**DOI:** 10.1136/bmjopen-2015-010573

**Published:** 2016-02-22

**Authors:** Jon M Dickson, Louise H Taylor, Jane Shewan, Trevor Baldwin, Richard A Grünewald, Markus Reuber

**Affiliations:** 1 Academic Unit of Primary Medical Care, The University of Sheffield, Samuel Fox House, Northern General Hospital, Sheffield, UK; 2 The Medical School, The University of Sheffield, Sheffield, UK; 3 Research and Development, Yorkshire Ambulance Service NHS Trust, Wakefield, UK; 4 Emergency Operations Centre, Yorkshire Ambulance Service NHS Trust, Wakefield, UK; 5 Department of Neurology, Sheffield Teaching Hospitals NHS Foundation Trust, Sheffield, UK; 6 Academic Neurology Unit, The University of Sheffield, Sheffield, UK

**Keywords:** HEALTH SERVICES ADMINISTRATION & MANAGEMENT, NEUROLOGY, ACCIDENT & EMERGENCY MEDICINE

## Abstract

**Objectives:**

Suspected seizures are a common reason for emergency calls to ambulance services. Prehospital management of these patients is an important element of good quality care. The aim of this study, conducted in a regional ambulance service in the UK, was to quantify the number of emergency telephone calls for suspected seizures in adults, the associated costs, and to describe the patients’ characteristics, their prehospital management and their immediate outcomes.

**Design:**

Quantitative cross-sectional study using routinely collected data and a detailed review of the clinical records of a consecutive series of adult patients (≥16 years).

**Setting:**

A regional ambulance service within the National Health Service in England.

**Participants:**

Cross-sectional data from all 605 481 adult emergency incidents managed by the ambulance service from 1 April 2012 to 31 March 2013. We selected a consecutive series of 178 individual incidents from May 2012 for more detailed analysis (132 after exclusions and removal of non-seizure cases).

**Results:**

Suspected seizures made up 3.3% of all emergency incidents. True medical emergencies were uncommon but 3.3% had partially occluded airways, 6.8% had ongoing seizure activity and 59.1% had clinical problems in addition to the seizure (29.1% involving injury). Emergency vehicles were dispatched for 97.2% of suspected seizures, the seizure had terminated on arrival in 93.2% of incidents, 75% of these patients were transported to hospital. The estimated emergency management cost per annum of suspected seizures in the English ambulance services is £45.2 million (€64.0 million, $68.6 million).

**Conclusions:**

Many patients with suspected seizures could potentially be treated more effectively and at lower cost by modifying ambulance call handling protocols. The development of innovative care pathways could give call handlers and paramedics alternatives to hospital transportation. Increased adoption of care plans could reduce 999 calls and could increase the rates of successful home or community treatment.

**Strengths and limitations of this study**

- This is the first published study to report the clinical details and outcomes of patients for whom an emergency call to the ambulance service was made for suspected seizures.
- Although our sample of patients was relatively small, our results are consistent with other unpublished sources of these data and we think that they are likely to be generalisable throughout the National Health Service (NHS) in England.
- More research is required to confirm these results and to generate an evidence base to inform new models of prehospital care for management of people after a suspected seizure in the NHS and internationally.

## Introduction

### Emergency management and triage of suspected seizures

A single, uncomplicated self-limiting epileptic seizure in a patient with an established diagnosis of epilepsy usually only requires first aid, but suspected seizures are dramatic and may frighten observers, who frequently call emergency prehospital services for assistance.^1^ Suspected seizures may not be epileptic and many other conditions mimic epilepsy;^2^ therefore, we use the term ‘suspected seizure’ for this group of patients throughout this paper. Patients with suspected seizures present a significant diagnostic challenge, the cause of seizures and other paroxysmal events involving collapse, and loss of consciousness often remains obscure even after hospital admission. Emergency management decisions about these patients may be complex and require expertise, training and guidance. However, there is little evidence to inform best practice for these patients’ prehospital care.^3^ In the UK, accepted best practice guidelines are provided by the Ambulance Services’ Clinical Practice Guidelines.^4^

Status epilepticus (an epileptic seizure which does not stop spontaneously) is a medical emergency, and prognosis is dependent on the speed of treatment.^5^ There are other reasons why suspected seizures may require urgent medical intervention: they may be secondary to another medical problem such as cardiorespiratory arrest, hypoglycaemia, meningitis or another acute brain pathology, or the seizure itself may have caused an injury. Following suspected seizures, patients may be confused, emotionally upset and vulnerable.

The diagnostic heterogeneity of suspected seizures makes clinical triage of these emergency calls difficult. Ambulance services are under pressure to meet rapid response time targets, and many emergency vehicles are dispatched urgently for these patients. However, an appropriate balance between safety and cost-effectiveness is crucial, and rapid responses to suspected seizures may be unnecessary in many cases.

### Prehospital care and the ambulance services in the NHS in England

England, excluding the Isle of White, has a population of 42.96 million adults (52.96 million adults and children) and is served by 10 National Health Service (NHS) ambulance trusts.^6^ ^7^ This study was conducted using data from one of these organisations, the Yorkshire Ambulance Service (YAS). The fact that ambulance services in the UK are offered by a single provider in each region and not by competing emergency services means that UK ambulance service data provide epidemiological information which may be relevant, but more difficult to collect, in other countries. Each ambulance trusts covers a mean area of 5151 square miles (range 620–7500 m^2^) serving a mean population of 5.5 million (range 2.6–7 million).

In the UK, prehospital services are provided predominantly by paramedics and emergency care assistants (ECAs). Paramedics must undertake an approved university course or ambulance service training in paramedic science to achieve registration with the Health and Care Professions Council in order to practice. A paramedic is often the senior medical person at the scene of an incident and may be supported by an ECA. ECAs work under the supervision of a clinically qualified practitioner, such as a paramedic, and are trained in emergency driving. Rapid response vehicles (RRVs) are cars staffed by a single paramedic, the cars are well stocked but do not have the full range of medical equipment carried by an ambulance. They are often first on the scene of an emergency incident and often avoid the need for an ambulance and two-person crew to attend. In other healthcare systems, it is common for emergency physicians to provide prehospital care but in the NHS this is rare. Helicopter Emergency Medical Services (HEMS) are staffed by emergency physicians but these would only very rarely be dispatched for suspected seizures.

### Emergency call handling

Emergency calls to YAS are handled in two emergency operations centres (EOCs) by specially trained call handlers. Six out of 10 UK ambulance trusts use the Advanced Medical Priority Dispatch System (AMPDS; the other trusts use NHS pathways) to prompt call handlers to ask standard questions aimed at rapid and accurate assessment of the speed and type of response required. AMPDS is based on 33 protocols tailored to a range of clinical presentations. Protocol 12 is used by ambulance call handlers for suspected seizures.

Once triaged, the dispatch of an emergency vehicle is coordinated using the computer aided dispatch (CAD) system to ensure that patients are attended by the most appropriate staff and vehicle in a timely fashion. The callers’ responses to questions in the AMPDS protocols yields ‘determinant descriptors’ (such as ‘continuous or multiple fitting’ or ‘effective breathing not verified’) which are used to assign priorities, target response times (described later in this paper) and the type of response. Response options include telephone advice, dispatch of emergency vehicles (ambulance or RRV), dispatch of community responders, referral to other services (general practitioners (GPs), social services, district nurses) and dispatch of specialist resources.

### Aims and objectives

This study, Epilepsy Pre-hospital Interventions and Care (EPIC 1), was designed to document the clinical characteristics of patients managed by ambulance services with suspected seizures, describe their prehospital management, their immediate outcomes and to estimate the cost of their emergency care. EPIC1 is part of a programme of research intended to develop innovative models of care that could improve quality and cost-effectiveness of emergency care for suspected seizures in the NHS and other healthcare systems internationally.^8–10^

## Methods

### Setting

The patients described here were identified from the records of YAS, a regional UK ambulance service covering 6000miles^2^ (9656 km^2^) and serving a population of 4 954 876 people (4 019 610 adults).^6^ YAS has 2 EOCs, 62 ambulance stations, 19 hospital-based patient reception centres and a fleet of over 500 emergency vehicles.^11^ Its annual budget is approximately £231 million (€327 004 763, $351 167 355). Its boundary encompasses 20 contracted clinical commissioning groups (CCGs; doctor-led organisations who commission health services for the people within their boundaries). Sheffield is one of the major urban centres within the area served by YAS; the Sheffield CCG area has a population of 451 100 adults (551 756 adults and children) and is served by a single hospital-based emergency department.^7^

### Data

Routinely collected YAS data were extracted from the CAD system for incidents managed between 1 April 2012 and 31 March 2013 in adults (≥16 years old). These data were categorised by AMPDS protocol. CAD data regarding geographical, diurnal and monthly variation of incidents were obtained as well as data about the timing of each stage of the emergency call. These timings are automatically recorded and labelled to allow assessment against performance criteria. T0 is the time the 999 call is received and subsequent key events within the call are labelled T1–6. In terms of dispatch of vehicles, Ta (allocation) is the time that the incident is allocated to a vehicle and Tr (response) is the time that the vehicle arrived on the scene.

We combined our analysis of the YAS-wide data with a detailed retrospective review of emergency calls about suspected seizures from Sheffield focusing especially on a consecutive series of 178 suspected seizure incidents that occurred in May 2012 within the Sheffield CCG boundary. The sample period of May 2012 was chosen after preliminary analysis of the summary statistics showed it to be a typical month in terms of call volume. There was no reason to suspect that this sampling strategy introduced any bias.

The Patient Report Form (PRF; ambulance clinicians’ clinical notes) for each suspected seizure was retrieved. Patient identifiable data were redacted by YAS, and anonymised copies of the PRFs were supplied to the research team. Each PRF was analysed using a data extraction tool which was developed by the authors and revised after an initial pilot study. All the data were analysed using standard descriptive statistics. The numerator and denominator are shown for each percentage cited in this paper, so that readers can see if it pertains to the whole series of 178 incidents (for which CAD data were available), the series after exclusions (154/178), the series after non-seizure incidents had been removed (132/178) or another subgroup from the series. Throughout this paper, we report data as it was recorded in the notes by the clinicians involved in the case. In addition, using the information available from the PRF, the suspected seizures were classified by two neurologists with an interest in seizure disorders (MR and RAG). ECGs (3 lead or 12 lead), when available, were categorised into normal (sinus rhythm, 60–100 bpm) or abnormal.

#### Costs

Ambulance service costs are based on individual agreements between the individual ambulance services and the contracting CCGs (who negotiate collectively with their local ambulance service). Ambulance services have three tariff bands for managing incidents and YAS tariffs were obtained: £45.43 for managing an incident exclusively with telephone advice (hear and treat/refer), £200.76 for dispatch of an ambulance or RRV without transport to hospital (see and treat/refer) and £221.57 for dispatch of an ambulance or RRV plus transport to hospital (see and treat and convey). These tariffs are applied regardless of the urgency of the ambulance response. The total cost of their activity for managing the series of incidents in the study was calculated.

## Results

### Routinely collected data

#### Summary data from Yorkshire and Sheffield

Between 1 April 2012 and 31 March 2013, YAS dealt with 605 481 emergency incidents in adults (≥16 years old) within the boundaries of its contracted CCGs (plus 49 912 incidents in children; it also dealt with an additional 2434 incidents from outside of the boundaries of these CCGs which are not included in this analysis). Throughout the rest of this paper, the term ‘incidents’ refers to adult incidents (excluding children). Over the course of the year, 19 799/605 481 (3.3%) incidents were suspected seizures which makes this category the seventh most common single-issue call type throughout YAS (see table 1). The number of suspected seizure incidents varied between the CCGs with a mean of 5.2 per 1000 population per year (SD 2.3, range 2.9–13.7).

**Table 1:** Ranking of the most common AMPDS single issue protocols assigned to incidents within YAS and within the Sheffield CCG during 2012/2013

Rank	AMPDS protocol name (number)	Sheffield	Sheffield (%)	YAS	YAS (%)
1	Falls (17)	9476	12.8	78 491	13.0
2	Chest pain (non-traumatic) (10)	7105	9.6	64 789	10.7
3	Breathing problems (6)	5447	7.3	49 351	8.2
4	Sick person (specific diagnosis) (26)	5658	7.6	41 675	6.9
5	Unconscious/fainting (near) (31)	4463	6.0	37 458	6.2
6	Stroke—CVA	2177	2.9	20 277	3.3
7	Convulsions/fitting (12)	2121	2.9	19 799	3.3
8	Haemorrhage/lacerations (21)	2389	3.2	19 001	3.1
9	Overdose/poisoning (ingestion) (23)	2222	3.0	18 526	3.1
10	Abdominal pain / problems (01)	1843	2.5	15 106	2.5
Total (of top 10)			42 901	364 473	60.2
Total (all incidents)			74 141	605 481	–

This list excludes ‘protocol 35’ and ‘other’ incidents. Protocol 35 incidents and other incidents are handled differently to other emergency calls received by YAS. ‘Protocol 35’ is used for incidents called through by healthcare professionals regardless of the presenting complaint. ‘Other’ incidents are not coded using AMPDS, for example, incidents that were initially triaged to the clinical hub for telephone management and subsequently passed back to the dispatcher to receive an ambulance response.

AMPDS, Advanced Medical Priority Dispatch System; CCG, clinical commissioning group; CVA, cerebrovascular accident; YAS, Yorkshire Ambulance Service.

#### Data from Sheffield

There were 74 141 incidents in 2012/2013 originating from within the Sheffield CCG boundary of which 2121 (2.9%) were suspected seizures. The highest density of calls was around the city centre and the call density was much lower in rural locations. The mean number of calls per month for suspected seizures was 176 (SD 13.6). In Sheffield, in May 2012, there were 6107 emergency incidents of which 178 (2.9%) were suspected seizures. There was a diurnal variation in call volume that was similar to the diurnal variation for the total number of calls for all causes. There is a rapid rise in call volumes from the nadir at approximately 5:00, to a plateau 12:00–21:00, which then slowly drops back to the nadir at 5:00.

### Data from Sheffield in May 2012

#### Exclusions, demographics, medial history and drug history

**Figure 1:**
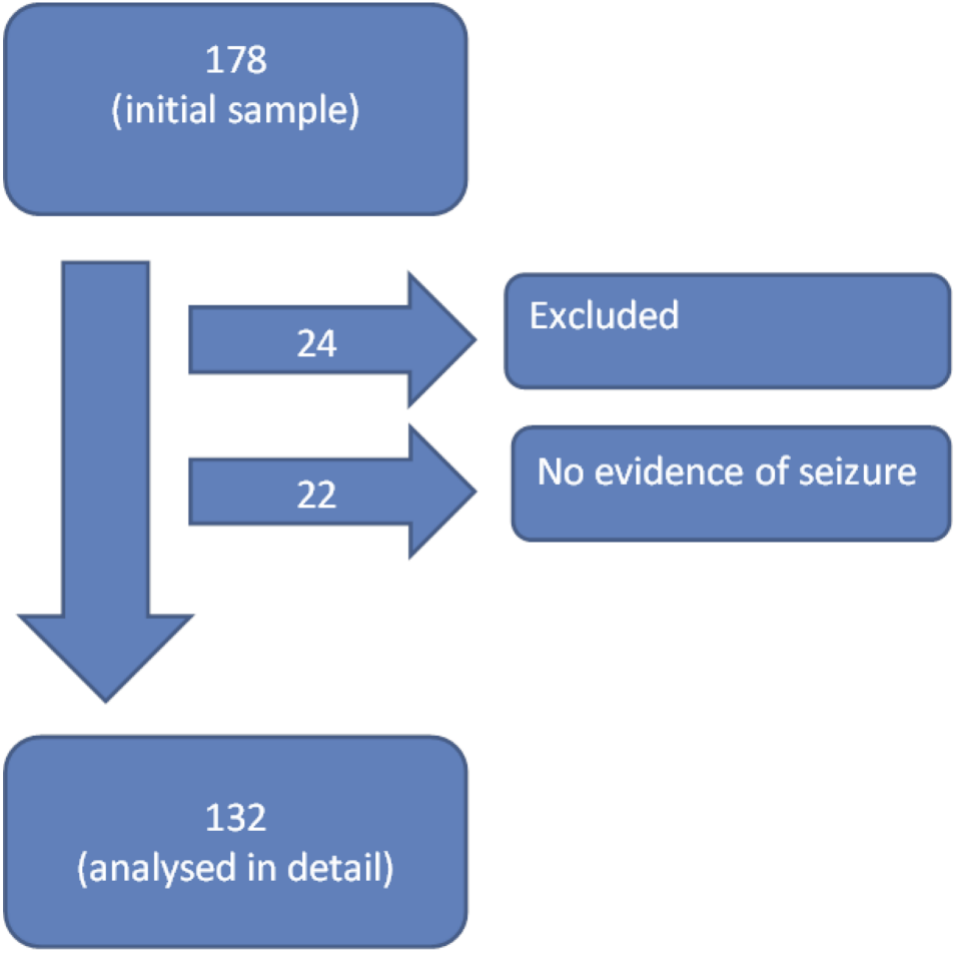
Flow chart to show how the series of 132 incidents which were analysed in detail were derived from the initial sample of 178 incidents. In total, 24/178 incidents were excluded: missing/inadequate data (18/178; 10.1%) and miscellaneous, for example, hoax call (6/178; 3.4%). The clinical impression of the ambulance clinicians was that there was no evidence of seizure activity in 22/178 (12.4%) incidents. The recorded diagnoses in these cases were syncope (3), intoxicated/passed out (2), tremor/spasm (2), fall (2), rigours (2), twitching (1), panic attack (1), anxiety/hyperventilation (2), abnormal behaviour (1), social/miscellaneous/inappropriate (6).

After exclusions, and after removing non-seizure cases, 132 incidents were analysed in detail (figure 1). The 132 incidents that were included in the final data analysis pertained to 124 individual patients; 8 patients generated two incidents each during the data collection period. The interval between the repeat incidents was ≤24 h (4), 3 days (1), 4 days (1), 13 days (1) and 14 days (1). The median age of the patients was 41 years (IQR 28, range 16–97), 57.6% were male and 42.4% were female. The medical history and drug histories in the PRFs documented that 56/132 (42.4%) had a history of epilepsy, 3/132 (2.3%) had a diagnosis of psychogenic non-epileptic seizures and 1/132 (0.8%) had a history of both epilepsy and psychogenic non-epileptic seizures. There was a documented history of antiepileptic drug (AED) use in 45/132 (34.1%). This was AED monotherapy in 27/45 (60.0%) and AED polytherapy (usually with just two AEDs) in 8/45 (17.7%). In 8/45 (17.7%) non-concordance with a recommended drug regimen was reported.

#### Location of incidents

The incidents took place in a private residence in 79/132 (59.8%), a public place such as a supermarket in 42/132 (31.8%) and a place of work/education in 7/132 (5.3%). The relationship of the caller to the patient could be established in 65/132 (49.2%) of incidents. The most common people to call 999 on behalf of the patient were a relative/friend/carer (45/65; 69.2%) or a member of the public (13/65; 20.0%).

### Call handling, resources, response times and determinant descriptors

An emergency ambulance and/or RRV was dispatched to 173/178 (97.2%) incidents in the first instance (a vehicle may have been subsequently dispatched to the remaining 5 incidents). For the series of 132 incidents where PRFs were reviewed in detail, the median time between the emergency telephone call being received (T0) and an ambulance being dispatched (Ta) was 41 s. The median time at which the call handler entered a specific APMDS protocol (T5; ie, made their preliminary diagnosis after asking basic questions) was 125 s. This means that ambulances were automatically dispatched an average of 84 s before the ambulance call hander had made their preliminary diagnosis. In 62/132 (47.0%) of cases, both an RRV and an ambulance were dispatched and arrived on the scene, in 53/132 (40.2%) only an ambulance attended and in 17/132 (12.9%) only an RRV attended. In total, 194 emergency vehicles were dispatched to the 132 incidents. The mean time on scene (either RRV, ambulance or both) for patients that were transported to hospital was 27 min (SD 15, range 3–101).

An emergency vehicle was dispatched with an 8 min response time (the highest priority response available to the emergency call handlers) in 73/132 (55.3%) incidents, 49/132 (37.1%) generated an emergency vehicle dispatch with a 20 or 30 min response time and 5/178 (2.8%) were dealt with by telephone (hear and treat). Four determinant descriptors encompassed the majority (168/178, 94.4%) of incidents (see table 2): ‘continuous or multiple fitting’ (12-D-02; 76/178, 42.7%), ‘effective breathing not verified ≥35’ (12-D-04; 30/178, 16.9%), ‘effective breathing not verified <35’ (12-B-01; 15/178, 8.4%), ‘not fitting now and breathing effectively verified’ (12-A-01; 47/178, 26.4%).

**Table 2:** Determinant descriptor codes for the suspected seizure incidents in May 2012 within the Sheffield CCG, their call category and their associated target response times

Determinant descriptors	Determinant code	Call category	Response times	Incidents (e)	Self-terminated	Ongoing	Subsequent
Not breathing (after key questioning)	12-D-01	Red 1 (A)	Response in 8 min	00	–	00	00
Continuous or multiple fitting	12-D-02	Red 2 (A)	Response in 8 min	76 (51)	51/59 (86.4%)	08	03
Agonal/ineffective breathing	12-D-03	Red 2 (A)	Response in 8 min	00	–	00	00
Effective breathing not verified ≥35	12-D-04	Red 2 (A)	Response in 8 min	30 (17)	20/20 (100%)	00	00
Focal fit (not alert)	12-C-01	Red 2 (A)	Response in 8 min	01 (01)	1/1 (100%)	00	00
Pregnancy	12-C-02	Red 2 (A)	Response in 8 min	01 (00)	1/1 (100%)	00	00
Diabetic	12-C-03	Green 1 (C)	Response in 20 min	03 (02)	2/2 (100%)	00	00
Effective breathing not verified <35	12-B-01	Green 1 (C)	Response in 20 min	15 (10)	12/13 (92.3%)	01	01
Not fitting now and breathing effectively (verified)	12-A-01	Green 2 (C)	Response in 30 min	47 (31)	35/35 (100%)	00	02
–	–	Green 3 (C)	Tel assess within 20 min		–	–	–
Focal fit (alert)	12-A-02	Green 4 (C)	Tel assess within 60 min	02 (01)*	–	00	00
Impending fit (aura)	12-A-03	Green 4 (C)	Tel assess within 60 min	03 (03)*	1/1 (100%)	00	00
				**178**	**123/132**	**9/132**	**6/123**

The data in the incidents column pertain to the whole series of 178 incidents. The data in the subsequent three columns pertain to the 132 incidents that were analysed in detail (after exclusions). Incidents=number of incidents, (e)indicates the number in each category who answer yes to the protocol 12 question ‘is s/he an epileptic or ever had a fit before?’. Tel assess=telephone assessment. Ongoing=seizure ongoing on arrival of the clinicians. Subsequent=seizure had self-terminated on arrival of the clinicians but a subsequent seizure occurred. Telephone assessment for incidents in green 3–4 are dealt with using the PSIAM system which is a computerised set of clinical algorithms used by clinicians in the Clinical Hub. The clinical hub is staffed by healthcare professionals which includes nurses, paramedics and other specialists, these staff have received specialist training from YAS to provide telephone clinical assessment and advice to a selected groups of patients and operational ambulance staff. Telephone response was via NHS Direct.

CCG, clinical commissioning group; NHS, National Health Service; PSIAM, Priority Solutions Integrated Access Management; YAS, Yorkshire Ambulance Service.

### Clinical details

In 9/132 (6.8%) incidents, seizure activity was ongoing on arrival of the ambulance clinician. In 2/9 of these incidents, the seizure ceased and then recommenced and in 4/9 the seizure ceased during the attendance and there was no further activity, and in 3/9 there was continual seizure activity through attendance. In 123/132 (93.2%) incidents, the seizure had stopped on arrival of which, 6/123 had a subsequent seizure during the attendance and 117/123 had no subsequent seizures during the attendance. In 48/132 (36.4%) incidents, the patient was alert on arrival of the ambulance clinician and in 61/132 (46.2%) incidents the patient was described as ‘postictal’ by the ambulance clinician. In 14/132 (10.6%) incidents, the clinical impression of the ambulance clinician was difficult to define from the PRF notes.

**Figure 2:**
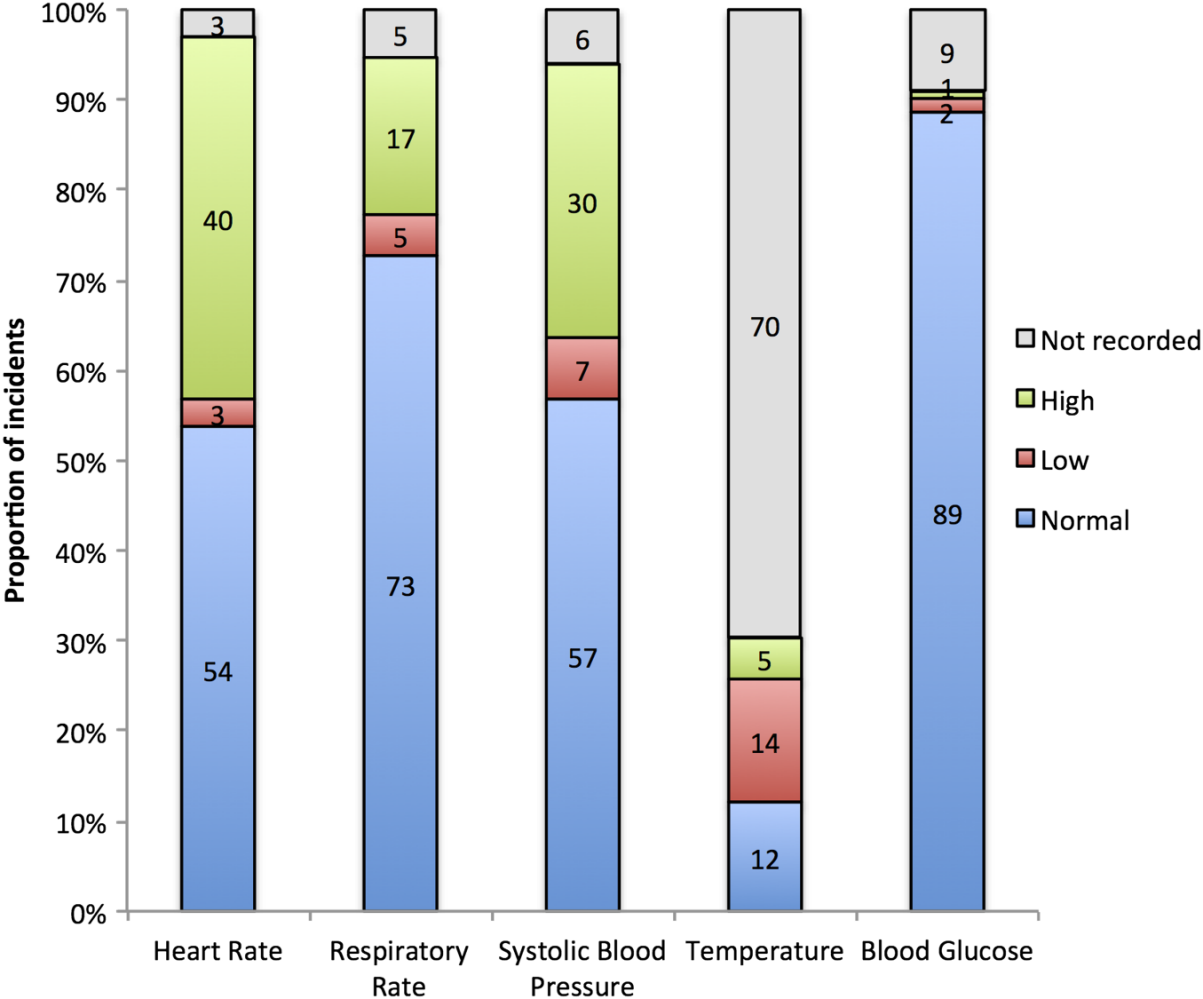
Percentages of normal and abnormal observations taken on arrival of the ambulance clinician(s). The numbers on the graph represent the percentage (rounded to whole numbers) of the 132 incidents, which were analysed in detail. Normal values used are: heart rate 60–100 bpm, respiratory rate 14–18 breaths/minute (males); 16–20 breaths/minute (females), systolic blood pressure 100–140 mm Hg, temperature 36.5–37.5°C, blood glucose 3.5–11.1 mmol/L.

Cardiac arrest had occurred in 0/178 (0%) incidents. The airway was clear in 120/124 (96.7%) and at least partially occluded in 4/124 cases (3.3%) (there were missing data for 8 incidents). Breathing was normal in 119/124 (96.0%) and abnormal in 5/124 (4.0%). Figure 2 shows the observations on arrival of the ambulance clinician. In 69/132 (52.3%), an ECG was performed which was abnormal in 14/69 (20.3%). Sinus tachycardia accounted for 50% (7/14) of abnormal ECGs. In 56/132 (42.4%) of incidents, there was one clinical issue in addition to the seizure such as an injury or a second medical problem; in 19/132 (14.4%), there were two additional clinical issues, and in 3/132 (2.3%), there were three additional clinical issues. The 103 additional clinical issues were varied and often created a complex clinical scenario. Alcohol was mentioned in 35/103 (34.0%), and 30/103 (29.1%) of the clinical issues were injuries. Other clinical issues included brain tumour, symptoms of acute coronary syndrome, severe hypertension, illicit drug use, stroke and hypoglycaemia. There were a wide range of injuries, some minor, but many were more severe such as facial cuts, dental trauma, scalp haematomas, shoulder dislocations and many patients had multiple injuries. Pregnancy was not documented in any incidents. The suspected seizures were classified by two consultant neurologists as follows: generalised tonic-clonic (66/132, 50.0%), focal (4/132, 3%), inadequate description (21/132, 15.9%), unclassifiable (34/132, 25.8%) and non-epileptic attack (7/132, 5.3%). An assessment of mental capacity was documented in 119 incidents. In total, 94/119 (78.9%) were deemed to have capacity, and 25/119 (21.0%) were deemed not to have capacity.

### On scene management

Airway support was used in 6/132 (4.5%) of incidents. This was an oropharyngeal airway in 4/6 incidents and an oropharyngeal airway plus suction in 1/6 incident. One (1/6) incident required an oropharyngeal airway, a nasopharyngeal airway and suction. Oxygen was administered in 29/132 (22.0%) cases. Intravenous cannulation was attempted in 18/132 (13.6%) of incidents and often required multiple attempts, 8/17 (47.1%) of successful cannula insertions were actually used by an ambulance clinician. In 18/132 (13.6%) incidents, the ambulance clinician contacted another service or professional during the incident. The majority of these (11/18) were hospital prealerts by telephone. Other external agencies contacted included the police, nursing staff and supported accommodation staff. In 11/132 (8.3%) incidents, emergency drugs were administered to terminate the seizure. In 3/11, drugs (clobazam and midazolam) were administered by a carer prior to arrival of the ambulance clinicians (none of these patient received a second dose). In 8/132 cases, emergency treatment was administered by an ambulance clinician. In total, 6/132 received a single drug (4 intravenous diazepam, 2 PR diazepam), and 2/132 received two drugs (1 intravenous diazepam twice, 1 PR diazepam followed by intravenous diazepam).

### Outcome

The ambulance clinicians advised transport to hospital in 118/132 (89.4%) incidents and advised that patients were left at the scene in 5/132 (3.8%) incidents. The majority of patients, 98/132 (74.0%), were transported to hospital and 27/132 (20.5%) were left at the scene. There were some significant discrepancies between the advice of the ambulance clinicians and the actual destination of the patient. The most important discrepancy was in patients who were advised to go to hospital but were actually left at the scene (or who were transported home or walked-off) which applied to 19/118 (16.1%) incidents. The Clinical Practice Guidelines^4^ provide four criteria to guide decisions about suitability for home management: known epilepsy, full recovery, not at risk, adequate supervision. When these were applied to all 132 incidents, 9/132 (6.8%) would have met all four criteria.

There was a referral to services other than a hospital emergency department (ED) in 4/132 (3.0%) incidents. Of the patients who were not transferred to hospital, 23/33 (69.7%) were given advice prior to departure of the ambulance clinician(s): 16/33 were advised to call 999 again if required, 8/33 were told to contact their GP, 2/33 were advised to contact an epilepsy specialist nurse. In 21/25 incidents where the patient was deemed not to have capacity, they were taken to hospital and in 3/25 they were left at the scene.

### Costs

In 2/162 (1.2%) incidents, telephone advice only (hear and treat/refer) was provided, 30.2% (49/162) were managed by dispatch of an emergency ambulance without transport to hospital (see and treat/refer) and 68.5% (111/162) by dispatch of emergency ambulance plus transport to hospital (see and treat and convey). Cost data were missing for 16/178 incidents, so these costs were extrapolated to the whole series of 178 incidents. The total costs for each category of incident were: £90.86 ((0.012×178)×£45.43), £10 841.04 (0.302×178)×£200.76) and £27 031.54 ((0.685×178)×£221.57) respectively. The estimated total cost of managing the entire series of 178 suspected seizures was therefore £37 963.

The total cost of £37 963 for managing our series of 178 suspected seizure incidents from Sheffield was used to estimate annualised costs in Sheffield, YAS and England. Our study provided actual data for the number of incidents per year in Sheffield (2121) and YAS (19 799). The number of incidents per year in England was calculated using the YAS average of 4.93 per 1000 of the adult population (42 960 000/1000×4.93) which equals 211 793. Therefore, the estimated annualised ambulance service costs for managing suspected seizures in each geographical area are: Sheffield £452 357 (2121/178×£37 963) (€639 772, $686 614), YAS £4 222 637 (19 799/178×£37 963) (€5 978 046, $6 416 089), England £45 170 212 (211 793/178×£37 963) (€63 966 441, $68 663 386).

## Discussion

### Call handling, triage and resource allocation

Our data show that the majority of suspected seizures are rapidly self-limiting and only a minority of patients require immediate medical treatment. Self-termination of the suspected seizure and resumption of verifiable breathing occur before the emergency vehicle arrives in most cases. Only 8.3% of our sample required emergency drugs for seizure termination, and there were no cases of cardiac arrest. Despite this, 97.2% of the 178 incidents in the series were dealt with by dispatch of an ambulance or RRV. One hundred and ninety-four vehicles were dispatched for the 132 incidents that were studied in detail. Most calls were given the highest priority, with a response time target of 8 minutes. Only five calls were managed with telephone advice.

AMPDS is an international system that is central to clinical triage in the majority of UK ambulance services. It is configured to ensure that a rapid response is sent to patients in whom breathing cannot be verified because this may be a manifestation of cardiac arrest (or other non-perfusing cardiac rhythms) and may need rapid delivery of cardiopulmonary resuscitation (CPR) ±defibrillation.^12^ ^13^ Other studies have shown the incidence of cardiorespiratory arrest in calls categorised as suspected seizures to be 0.36–2.1%.^14^ ^15^ The current system of rapid dispatch based on the initial entries of call handlers into CAD fields allows response time targets to be met. In practice, this means ambulances and RRVs being dispatched, based on free-text entries into CAD, before the diagnosis is clear. In this study, the median time between the emergency telephone call being received (T0) and that of an ambulance being dispatched (Ta) was 41 s. This was an average of 84 s before the ambulance call hander had made their preliminary diagnosis (T3) (125 s) and entered the AMPDS algorithm for suspected seizures. This method may not be the best use of scarce resources.

Differentiating between an epileptic seizure and a cardiorespiratory arrest is problematic because effective breathing also ceases during generalised tonic-clonic (GTC) seizures (the most common type of epileptic seizure leading to emergency calls), and breathing is often difficult to verify during other types of epileptic seizure. There are little data on the duration of epileptic seizures, but it has been shown that most secondary generalised tonic-clonic seizures last <2 min and that epileptic seizures lasting >5 min are uncommon.^16^ ^17^ While immediate postictal respiration is often abnormally noisy and individual breaths are unusually deep, in most cases regular inspiration and expiration resumes rapidly after GTC seizures.^18^ Therefore, if call handlers held the line for, for example, 3 min after making the decision to dispatch, they would be likely to be able to confirm normalisation of breathing in many cases which may allow a greater refinement of dispatch decisions and allow more cost-effective care to be delivered.

It has recently been suggested by NHS England,^19^ that slightly delaying the decision to dispatch emergency vehicles may be safe and cost-effective. Such a strategy may have no effect on the outcomes of the majority of suspected seizure incidents, but it may adversely affect the outcomes of the true medical emergencies in this group including those in cardiac arrest^15^ and status epilepticus. The prognosis of these patients is dependent on the speed of delivery of effective treatment and careful cost-benefit analyses would be required to justify such an approach for patients with suspected seizures. Alternative strategies could include call handlers routinely staying on the line after making their decision to dispatch. This may allow many incidents to be stepped down in terms of priority once seizure termination and effective breathing are verified. Routinely dispatching only RRVs (rather than ambulances) to suspected seizures in the first instance may reduce the total number of vehicles dispatched per incident and improve cost-effectiveness.

### Transport to hospital and the potential for alternative care pathways

In total, 99/132 (75.0%) of our sample of patients were transported to hospital and only 20.5% (27/132) were left at the scene. This rate of transport to hospital is consistent with the only other sources of this type of data that we are aware of.^20^ ^21^ Follow-up data from ED and inpatient wards is required to draw definitive conclusions about what proportion of patients required hospital assessment and treatment, but our data suggest that a higher proportion would be suitable for home management if paramedics felt appropriately supported. Current UK ambulance service guidelines state that “…known epileptics who make a full recovery, are not at risk and can be supervised adequately, can be managed at home following local guidelines.”^4^ This statement is the only guidance that is provided and it does not encourage or support community management. UK paramedics do not feel competent to decide if a patient is safe at home after a suspected seizure and they do not have access to care pathways that facilitate urgent follow-up.^21^ A recent qualitative study of decision-making in 15 emergency ambulance clinicians in the UK identified themes such as lack of experience, patient views, insufficient information, anxiety over litigation, lack of access to the patients’ medical records and bystander expectation, as factors influencing the rate of transfer to hospital in this group of patients. Many paramedics feel transport to hospital is both clinically safer and a lower risk medicolegally.

Increased training^21^ and the development of a clinical guideline to support paramedic treatment and discharge (see and treat) with the option to make an urgent referral to a specialist via an alternative care pathways (ACPs) may avoid the need for the majority of patients to continue along the emergency care pathway to EDs and inpatient wards.^22^ ^23^ This would not only reduce ambulance service costs but also hospital costs both in terms of ED attendance and admission to hospital.^24^ An emergency call for an epileptic seizure often represents failed ambulatory care and it could be used as a trigger for urgent specialist review to prevent further seizures. The review could potentially be conducted by a neurologist, a physician with a special interest in seizures or an epilepsy nurse specialist. At the moment, many people with epilepsy are not appropriately followed up after a seizure in the NHS^25^ and lack of access to specialist services in many areas means suboptimal care for many patients.^26^ An ACP has the potential to generate significant cost savings and to improve patient care^27^ but very few ‘see and treat’ guidelines are in use across the UK ambulance services,^28^ uptake is poor for those that exist,^29^ and there have been no previous studies published on ACPs for suspected seizures.^30^ There are significant barriers to developing an ACP^22^ such as managing alcohol-related problems, safe community-based management of the postictal phase and community management of injuries^31^ and further research is required to support the development of effective interventions.

### Person-centred care and care planning

Despite the potential benefits of care planning, very few patients in the UK, including people with epilepsy, are involved in a collaborative care planning process.^26^ ^32^ ^33^ Our data show that a significant proportion of patients ‘refused transport’ after a suspected seizure which may indicate that they felt that hospital attendance was not required. A recent qualitative study looked at patients perspectives on emergency treatment for seizures.^1^ When seizures occur they are often witnessed by family members or a by-stander who are key determinants of attendance at ED (usually via 999 emergency calls). Although in retrospect many patients think that emergency treatment was not necessary, they feel that, given the circumstances, the person in question made the right choice to call an ambulance. A care plan for seizure management developed during routine ambulatory care has a lot of potential to reduce unscheduled accident and emergency attendances and hospital admissions. It could increase the confidence of patients, carers, bystanders and healthcare professionals to avoid emergency calls and to use ACPs that may reduce ED attendances and unscheduled admissions.

### Limitations

Our sample of 178 incidents was relatively small and our study was conducted in only one ambulance service. Although our data are consistent with the small number of other studies in this area and expert opinion, there may be limits to the generalisability of the data within the NHS and internationally. The sample was a consecutive series and random sampling throughout the year may have eliminated the small risk of bias inherent in the strategy actually used. Children <16 years old were excluded from the study because the aetiology of suspected seizures in children is significantly different to adults, management priorities are different and children have different health service providers to adults. Throughout this paper, we report data as it was recorded in the notes by the clinicians involved in the case. This does not imply that these data are accurate, for example, some diagnoses may have been made incorrectly, but we recorded the data in the data extraction tool verbatim with as little interpretation from the authors as possible. This study does not include definitive diagnostic/outcome data from medium and long-term follow-up and the clinical data available on the PRFs were limited—these are weakness of a retrospective study and a prospective study would allow stipulation of the variables to be collected. Finally, patients with seizures may be triaged to other AMPDS protocols (especially ‘unconscious/fainting’ AMPDS protocol 31 and ‘falls’ AMPDS protocol 7) which would not have been captured in this study, so our data may be an underestimate of the incidence of this problem.

### Conclusions and further research

Despite its limitations, this study has shown that suspected seizures are one of the most important reasons for emergency calls to the ambulance service, and that the majority of these patients are transported to hospital despite a low prevalence of true medical emergencies. These results demonstrate the potential for improved and more cost-effective emergency management of suspected seizures. Further research is required to support service improvement, such as modification of dispatch decisions and the development of ACPs to facilitate urgent expert outpatient review without the need for transport to hospital EDs.
